# Application of C2 subfacetal screws for the management of atlantoaxial dislocation in patients with Klippel-Feil syndrome characterized by a narrow C2 pedicle and high-riding vertebral artery

**DOI:** 10.1186/s13018-022-03391-z

**Published:** 2022-11-16

**Authors:** Zhe Hou, Qiang Jian, Wayne Fan, Xingang Zhao, Yinqian Wang, Tao Fan

**Affiliations:** 1 Spine Center, Sanbo Brain Hospital, Capital Medical University, Beijing, People’s Republic of China; 2 Department of Neurosurgery, Beijing Luhe Hospital, Capital Medical University, Beijing, People’s Republic of China; 3 Faculty of Science, The University of British Columbia, Vancouver, BC Canada

**Keywords:** C2 subfacetal screw, Klippel-Feil syndrome, Congenital C2-3 fusion, Atlantoaxial dislocation

## Abstract

**Objective:**

This study aims to investigate the clinical application and feasibility of C2 subfacetal screws in patients with Klippel-Feil syndrome (KFS), narrow C2 pedicles, and high-riding vertebral arteries (HRVAs).

**Methods:**

The clinical data of seven patients with KFS, atlantoaxial dislocation, narrow C2 pedicles, and HRVAs treated with C2 subfacetal screws were analyzed in this retrospective study. The internal height, isthmus height, and pedicle width of C2 vertebra were measured using preoperative computed tomography (CT). Subfacetal screws were inserted for 7 patients (12 sides). The position and length of the screws were observed using postoperative CT. Intraoperative dura mater and vertebral artery (VA) injuries were recorded. Bone fusion was observed using follow-up CT.

**Results:**

The internal height was 10.5 ± 3.2 mm, the isthmus height was 3.7 ± 1.8 mm, the pedicle width was 3.0 ± 1.4 mm, and the screw length was 19.7 ± 1.5 mm. All patients had HRVAs and narrow pedicles. No injury to the dura mater and vertebral artery (VA) occurred in this group of patients. Bone fusion was achieved in all patients during follow-up.

**Conclusions:**

In patients with KFS, HRVA, and a narrow C2 pedicle, there is sufficient space below the C2 articular surface for screw insertion. When the pedicle is narrow and the C2 pedicle screw is not suitable for placement due to possible injury to the VA, subfacetal screws are a feasible alternative.

## Introduction

Klippel-Feil syndrome (KFS) refers to congenital cervical segmentation failure, which is a common bone structure deformity in the craniovertebral junction [12]. Common symptoms of the syndrome are short neck, low hairline, and limited head movement [7]. C2-3 is a common fusion zone that is often associated with the C0-1 fusion zone, which increases the stress on the atlantoaxial joint, leading to atlantoaxial dislocation (AAD) [10, 15]. Therefore, the stabilization of the atlantoaxial joint by fixation is the primary treatment strategy for patients with C2-3 fusion and AAD [4].

The pedicle screw is the most effective and commonly used fixation technique for the C2 vertebra [1]. A high-riding vertebral artery (HRVA) and a narrow pedicle are common factors that hinder the placement of C2 pedicle screws, and the incidence of HRVA is approximately 14.5% of the general population [26]. For patients with C2-3 fusion, the incidence of HRVA ranges from 54.8% to 60.8% [5, 22, 23, 25]. The pedicle of patients with C2-3 fusion often cannot accommodate 3.5 mm screws, and forced insertion of larger screws involves the risk of pedicle splitting, which easily damages the medial spinal cord and the lateral vertebral artery (VA). Furthermore, screws have to possess high biomechanical strength to bear the shear force after atlantoaxial reduction [24]. Thus, for patients with KFS, narrow C2 pedicles and HRVAs, choosing an appropriate and effective screw trajectory is difficult but necessary for a successful surgery.

Existing C2 screw alternatives include improved pedicle screws, lamina screws, and transarticular screws. However, the morphology of a C2-3 fusion is quite different from that of a normal C2 vertebra, and the aforementioned techniques cannot be used to fix the axis safely and effectively in some cases. In 2014, Patkar et al. reported the use of C2 subfacetal screws [18], which are effective in preventing VA injury. However, there are few reports of this technique.

This retrospective study analyzed the data of seven patients with KFS, with associated AAD, narrow pedicles, and HRVA, who underwent subfacetal screw insertion. Our study assessed the feasibility and effectiveness of C2 subfacetal screw insertion for these patients. The details of the clinical application and surgical technique of subfacetal screw insertion are reported.

## Methods

### Patient population

From June 2017 to April 2021, seven patients with C2-3 fusion with associated AAD, narrow pedicles, and HRVA presented to our department. These patients received C2 subfacetal screws insertion (12 sides), and all procedures were performed by the senior author of this study (F.T.). The clinical features of these patients are summarized in Table 1.

**Table 1:** Clinical characteristics and preoperative measurement of patients in this group

Patient	Sex/age	Main symptom	Radiographic abnormalities	Radiographic evaluation					
				Internal height		Isthmus height		Pedicle width	
				Left	Right	Left	Right	Left	Right
1	F/47	Gait disturbance	BI, AAD, KFS, AOZ	5.99	7.78	2.22	3.9	1.63	3.77
2	M/39	Limbs weakness	BI, AAD, KFS, AOZ	14.6	13.7	7.41	3.83	4.5	3.15
3	M/44	Limbs weakness and numbness	BI, AAD, KFS, AOZ	12.4	16.4	3.94	4.72	3.79	5.54
4	F/30	Limbs numbness	BI, AAD, KFS, AOZ	7.12	12.1	2.26	6.66	1.56	2.88
5	F/46	Headache and bucking	BI, AAD, KFS, AOZ	9	8.73	1.96	5.08	0	3.85
6	F/34	Limbs weakness and numbness	BI, AAD, KFS, AOZ	11.5	9.78	3.74	2.04	3.98	1.59
7	F/68	Limbs weakness, numbness, apsychia	BI, AAD, KFS, AOZ	6.58	11.90	1.85	2.54	2.82	3.06

*AAD* atlantoaxial dislocation; *AOZ* atlas occipitalization; *BI* basilar invagination; *KF* Klippel-Feil syndrome; *M* male; *F* female

### Radiological evaluation

Chamberlain line invasion and the atlantodens interval (ADI) were measured using midsagittal computed tomography (CT). Congenital AAD was diagnosed when the ADI was more than 3 mm; basilar invagination (BI) was diagnosed when chamberlain line invasion was more than 5 mm [8].

**Fig. 1:**
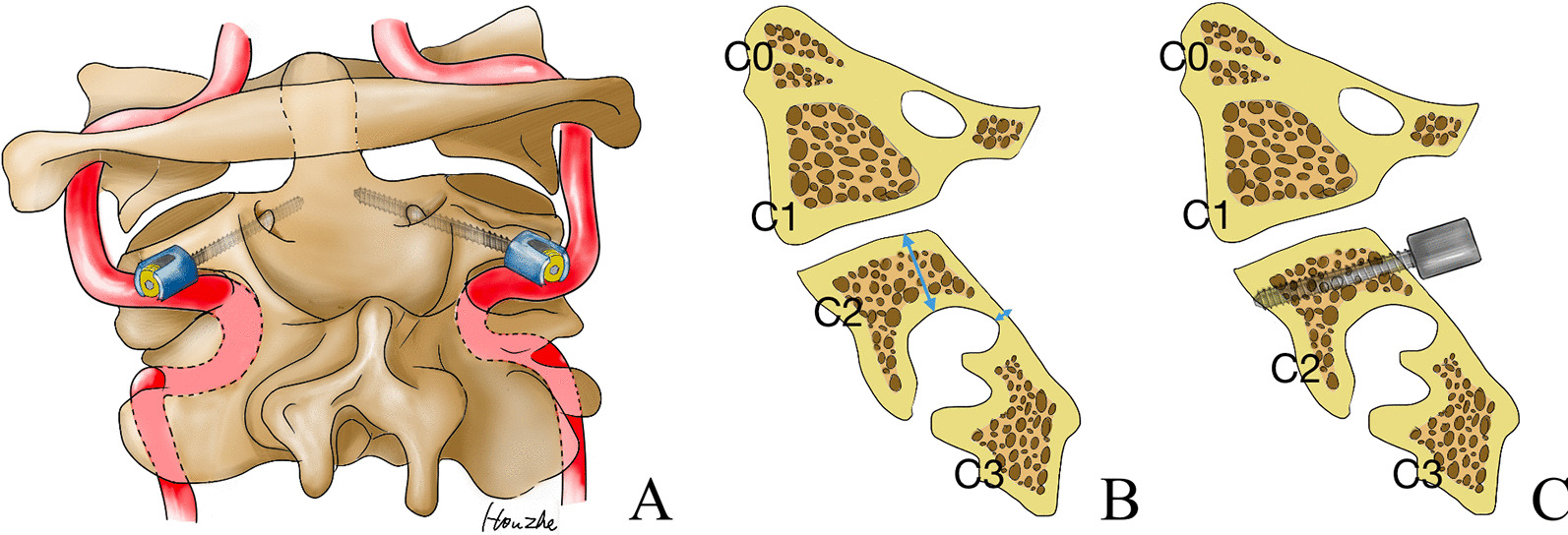
**A** Illustration of the C2 subfacetal screw for KFS. The selected entry point was approximately 3 to 4 mm below the midpoint of the posterior edge of the superior articular surface and parallel to the superior articular surface or slightly downward. The trajectory should be tilted inward as far as possible to obtain a longer stitching track. **B** Measurements of the internal height and isthmus height of patients with high-riding vertebral arteries (HRVAs). The isthmus height decreased significantly. **C** Illustration of the C2 subfacetal screw. The internal height provides sufficient space for the insertion of the C2 subfacetal screw

During preoperative parasagittal CT (sagittal view at 3 mm from the outside of the C2 spinal canal wall), we measured the internal height and the isthmus height of the C2 vertebra. The pedicle width was measured using axial CT. Narrow pedicle was considered when the maximum width of the pedicle did not exceed 4.0 mm [13]. HRVA was defined when the isthmus height did not exceed 5 mm and/or the internal height did not exceed 2 mm [16]. When the internal height was more than 4.0 mm, insertion of C2 subfacetal screws was considered safe (Fig. 1). Postoperative CT reconstruction was performed along with the screw trajectory and the screw length was measured.

### Surgical procedure

All patients in this study were treated with C1-2 joint distraction and occipitocervical fixation. They were placed in a prone position under general anesthesia, with the head fixed in the neutral position using the Mayfield headrest. The skin and subcutaneous layers were incised along the posterior median, exposing the squamous part of occipital bone superiorly and the C2 spinous process and vertebral lamina inferiorly. Thereafter, the C2 nerve root and venous plexus were lifted cranially under the microscope with a nerve stripper to reveal the lateral mass joint. Using the interfacet release and direct distraction technique described in previous research [21], the articular cartilage was removed using an ultrasonic osteotome. A cage with a suitable height was selected and filled with bone chips and artificial bone. Subsequently, the cage was placed in the interfacet space.

**Fig. 2:**
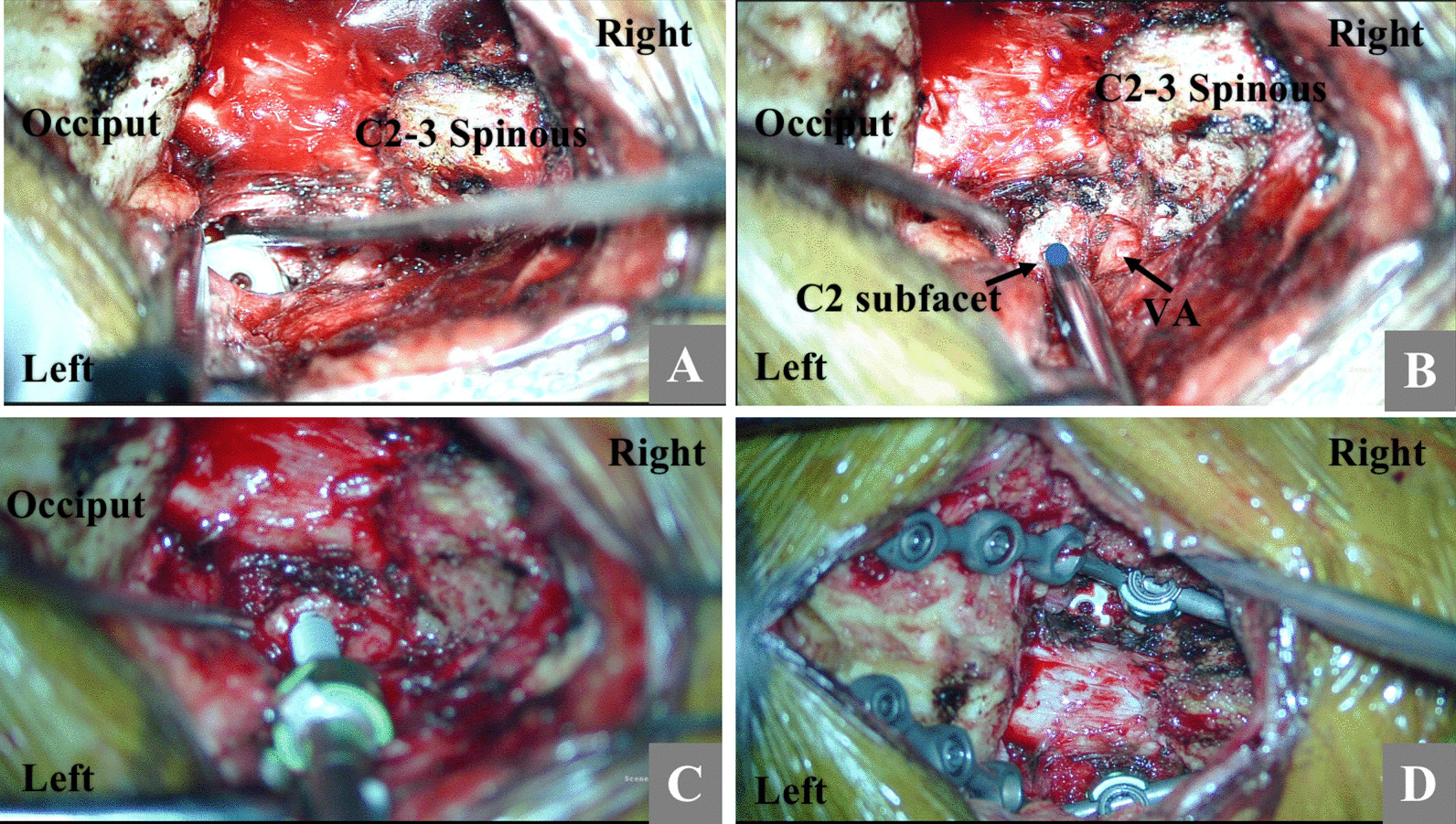
Intraoperative details of the placement of C2 subfacetal screws. **A** A cage was placed after bilateral lateral mass joints were released under the microscope, which increased the distance from the C2 superior articular process to the occipital bone, thus increasing the operative space for screw placement. **B** The screw entry point is located in the space above the vertebral artery and below the posterior edge of the C2 superior articular surface. The blue dot is the screw entry point of the C2 subfacetal screw. **C** The screw entry direction should be inclined to the inside as far as possible to enable insertion in the anterior vertebral body. **D** Finally, the titanium rods were shaped, and the occipital screws were connected

In patients with narrow pedicles, we chose to insert a C2 subfacetal screw. The chosen entry point was approximately 3 mm to 4 mm below the midpoint of the posterior border of the C2 superior articular surface, with the entry point parallel to the superior articular surface or slightly downward, with a medial inclination of approximately 20 degrees. Screws were inserted into the C2 vertebral body as far as possible. The chosen screw length was 22 mm or 24 mm and the depth of screw insertion was based on CT image measurements obtained before surgery (generally 17 to 21 mm) (Fig. 2). In patients without narrow pedicles, C2 pedicle screws and occipital screws were routinely inserted, followed by fixation with a rod. Finally, a supplementary artificial bone was placed in the space between the lateral articular surfaces bilaterally.

## Results

### Radiographic results

Patients in this study had an average internal height of 10.5 ± 3.2 mm and an average isthmus height of 3.7 ± 1.8 mm. All seven patients had a HRVA, caused by a reduction in the isthmus height without internal height decrease. The average pedicle width was 3.0 ± 1.4 mm, with 12 out of 14 sides having widths less than 4 mm (Table 1).

### Operative outcomes

In all the patients included in this study, C2 subfacetal screws were placed on 12 sides. The length of the subfacetal screws was 19.7 ± 1.5 mm. There was no spinal cord injury or VA injury during the operation (Table 2). During a mean follow-up period of 16 ± 5 months, screw fractures and cage displacement were not observed. In one patient, the screw was inserted into the transverse foramen during intraoperative screw insertion. We adjusted the screw direction inward and upward again. One patient experienced a postoperative incision infection that was successfully treated with debridement.

**Table 2:** Postoperative outcomes

Patient	C2 right	C2 left	Subfacetal screw length in bone	Postoperative neurological status	Transverse foramen violation	Bone fusion	Follow-up	Complications
1	Subfacetal	Subfacetal	18.2 (R) and 18.5 (L)	Improved	No	Yes	25	No
2	Subfacetal	Pedicle	21.3 (R)	Improved	No	Yes	22	No
3	Pedicle	Subfacetal	19.3 (L)	Improved	No	Yes	18	No
4	Subfacetal	Subfacetal	20.9 (R) and 21.2 (L)	Improved	No	Yes	17	No
5	Subfacetal	Subfacetal	21.5 (R) and 19.7 (L)	Improved	No	Yes	15	No
6	Subfacetal	Subfacetal	19.8 and 20.9 (L)	Improved	No	Yes	12	Infection
7	Subfacetal	Subfacetal	17.1 (R) and 17.9 (L)	Improved	Yes	Yes	15	No

*L* left; *R* right

## Discussion

C2-3 congenital fusion is often associated with AAD, which results in neurological dysfunction and requires reduction and fixation to relieve ventral cervical cord compression [10, 15]. Pedicle screws insertion is the commonly used technique for C2 fixation. Pedicle screws are combined with C1 lateral mass screws to perform Goel-Harms technique, which is a widely used fixation technique in clinical practice [3, 11]. The C2 pedicle screw can directly fix the isthmus and pedicle as a result of its long implant depth, good pull-out strength, and biomechanical stability [6]. However, C2-3 fused vertebrae are different from normal vertebrae, which are often associated with HRVA and narrow pedicles [22, 23].

**Fig. 3:**
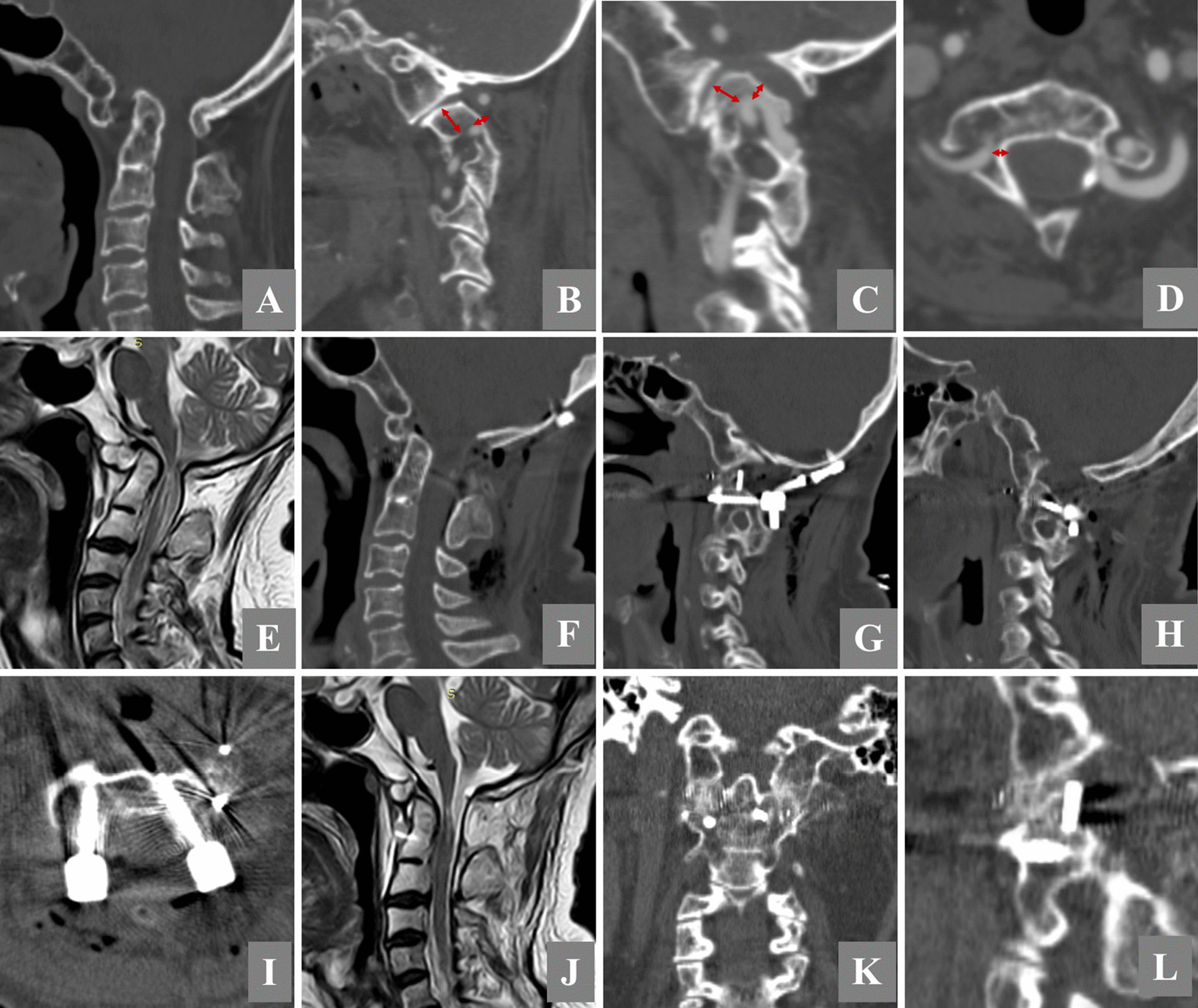
An illustrative case. A 36-year-old man presented with weak limbs and unstable motion. **A** Preoperative computed tomography (CT) showed an increased atlanto dental interval, C1 occipitalization, C2-3 fusion, bilateral atlantoaxial lateral joints tilted forward, an almost vertical left articular surface. **B**, **C** High-riding vertebral arteries on both sides. The medial height of the right side is 5.5 mm. The height of the isthmus is 3 mm. The medial height of the left side is 5 mm. The height of the isthmus is 2 mm. **D** Bilateral pedicle stenosis. The maximum width of the pedicle measured using axial CT is 2 mm. **E** A sagittal magnetic resonance (MR) T2 image shows atlantoaxial dislocation, the dentate process pressing the spinal cord, and a high signal in the spinal cord. **F** Sagittal CT showed a reduced atlantoaxial median joint and that the cage position between the lateral atlantoaxial joints was suitable (**G**, **H**). **I** Both screws are in proper positions. **J** Postoperative T2 MR showed that the spinal cord compression was relieved. There was still a high signal in the spinal cord. **K**, **L** CT scan showing bone fusion between the bilateral lateral joints at 8 months after surgery

Preoperative measurements of the patients in this study showed a decrease in the isthmus height and pedicle width of up to 3.7 ± 1.8 mm and 3.0 ± 1.4 mm, respectively. According to the diagnostic criteria for HRVA proposed by Neo et al.[16], namely, isthmus height 5 mm or less and/or internal height 2 mm or less, all seven patients (12 lateralities) had HRVAs. In this group of patients, the isthmus height was less than 5 mm (Fig. 3A, Table 1). The risks of inserting pedicle screws and atlantoaxial transarticular screws were high because of the reduced isthmus height. Moreover, the lack of the bony structure of the pedicle made it difficult to implant 3.5 mm pedicle screws. Therefore, it was necessary to select the appropriate entry point and screw trajectory.

Presently, commonly used alternatives to pedicle screws and transarticular screws include lamina screws, which are inserted through the junction of the spinous process and lamina. This technology is widely used, but when the height of the spinous process and the thickness of the axial lamina are small, its use is limited. Moreover, this technology only fixes the posterior single column structure and has a relatively weak biomechanical performance and a low fusion rate [14]. Rusconi et al. have reported the application of inferior articular process screws, but this technology only fixes the inferior articular process, which cannot achieve long trajectory length and thus a superior biomechanical property [19]. Furthermore, some studies have reported the use of transarticular screw fixation with the lower vertebrae [2], which is expected to achieve multi-cortical fixation through the facet joint. However, this technology fixes an additional vertebra and increases the fusion segments (C4) for patients with congenital C2-3 fusion, which worsens the cervical mobility of patients with C2-3 fusion. Some studies have reported improved pedicle screw techniques. Du et al. and Lee et al. have reported the medial “in–out-in” technique, which prevents VA injury by opening a window through the wall of the spinal canal to achieve multi-cortical tri-column fixation. Although no spinal cord injury has been reported, the screw entering the spinal canal poses a certain threat to the spinal cord [3, 13]. Additionally, Goel performed the VA transposition technique, using two smaller screws for fixation. However, this technique requires a careful separation of the VA, which is difficult to perform [9].

On the contrary, Patkar et al. and Salunke have used subfacetal screws for C2 screw placement [17, 20]. We found this technique particularly suitable for the patients in this study. Although the pedicles of C2-3 fused vertebrae are slender, the superior articular processes in patients in this study were thick. We measured 14 lateralities of seven patients in this group, and the average internal height was 10.8 ± 3.2 mm. The diameter of the commonly used screw is 3.5 mm, and it is assumed that the bone on both sides of the screw is at least 0.5 mm. Therefore, it is considered safe to place the subfacetal screws and pedicle screws in a bone with a minimum width of 4 mm. The average internal height of the C2 vertebra in the study patients was more than 4 mm and there was sufficient space for C2 subfacetal screw insertion (Fig. 1B). In addition, the entry point was located 3 mm to 4 mm below the articular surface in a small area above the transverse foramen (Fig. 1C). The screw trajectory was parallel to the articular surface or slightly downward, approximately 20 degrees inward, and tilted inward as far as possible into the vertebral body. The average effective screw length inserted in the study patients was 10.8 ± 3.2 mm. There was no incidence of nerve or blood vessel injury during the procedure. Additionally, there was no screw loosening or screw breakage observed during follow-up visits. A subfacetal screw in the vertebral body from the posterior facet of the articular process, achieving dual-column fixation, may be a better alternative to pedicle screws.

All patients in this study had AAD. Intrafacet distraction and implantation of the cage were performed during the procedure. The following details were given utmost attention during the procedure. First, the screw path of the C2 subfacetal screw is located on the upper and inner sides of the VA. If the screw loosens and subsidence occurs after surgery, VA injury would occur. Thus, a cage for inter-articular fusion was inserted to reduce the stress of screw and prevent subsidence. Second, because of the entry point of the C2 subfacetal screw close to the articular surface and the caudal tilted trajectory, the cage should be inserted first, and thereafter, the screw should be inserted. If the screw is inserted first, the path of the cage entering the lateral joint may be blocked by the screw head, which is not conducive for the preservation of the C2 nerve root. We observed a large operating space around the lateral articular space after cage implantation; therefore, there was no need to cut the C2 nerve root during the procedure. Third, examination images should be carefully studied before the procedure, with special focus on the internal height measurement and design of the screw path. The direction of the screw path should not be too downward to prevent the screw from entering the transverse foramen. The tapered tap should be carefully inserted during the puncture of the screw path. After inserting the tapered tap at 1 mm to 2 mm, the operator should retreat to observe the bleeding. After entering the tapered tap at a depth of 10 mm, it can be determined to exceed the vertebral artery, and then it is safe to advance the tapered tap. In one patient, a screw invaded the transverse foramen during the procedure, but no obvious bleeding was observed when the awl was used for puncture during the procedure; however, when the screw was inserted, it burst the screw path and partially entered the transverse foramen. We adjusted the screw placement direction inward and upward, and the vertebral artery was not damaged after screw placement.

**Fig. 4:**
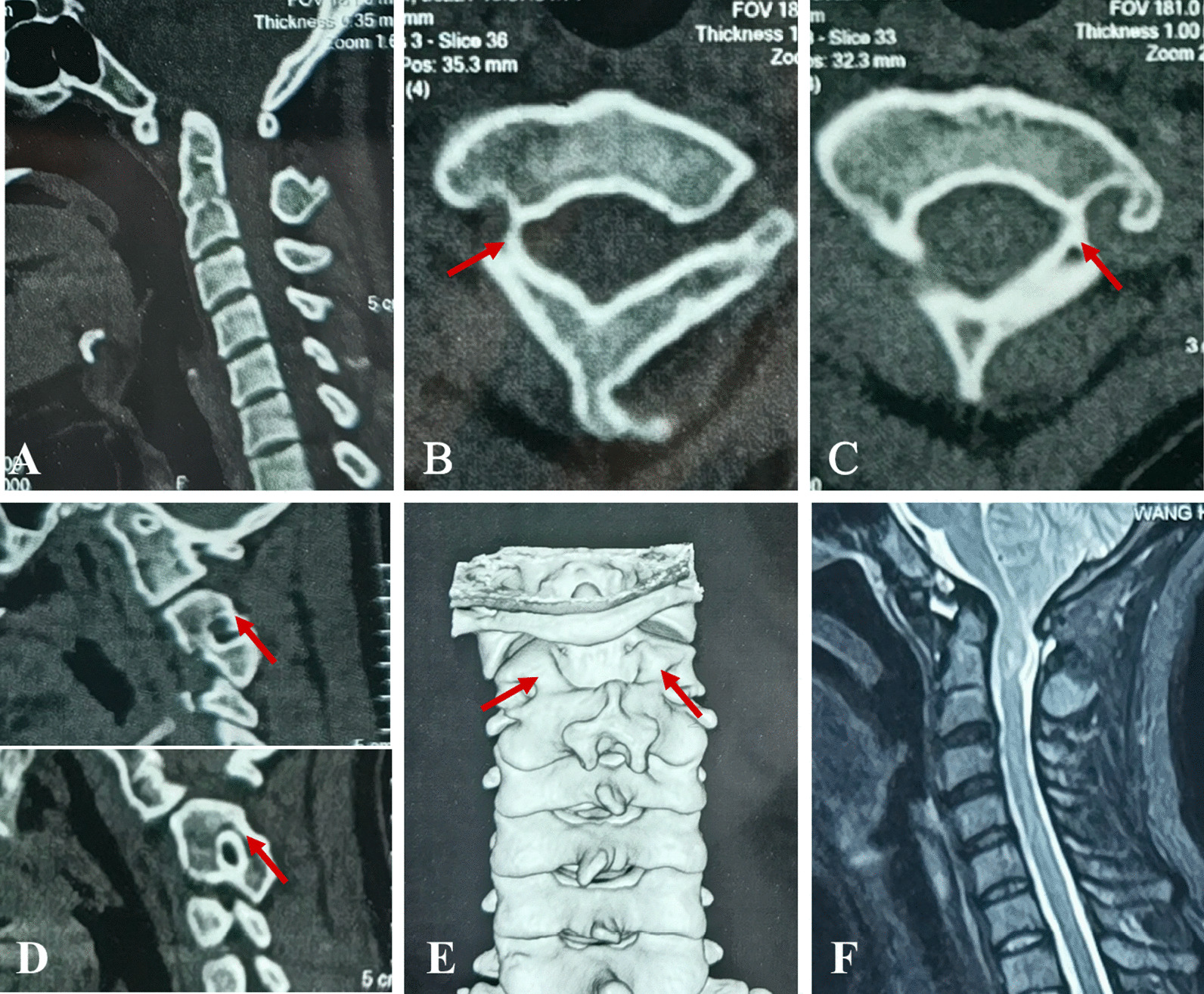
An illustrative case. A 33-year-old woman presented with occipital pain and unstable walking. **A** Preoperative sagittal computed tomography (CT) showed basilar invagination (BI), atlantoaxial dislocation, atlantooccipital fusion, and C2-3 fusion. Axial CT showed bilateral pedicle stenosis and the cortical pedicle (**B**, **C**). **D** The sagittal CT image shows a right internal height of 9.78 mm, isthmus height of 3.74 mm, left internal height of 11.5 mm, and isthmus height of 2.04 mm. Three-dimensional CT reconstruction showed that despite the high-riding vertebral arteries (HRVAs), there was still enough space above the vertebral artery to insert the subfacetal screw (**E**). **F** Sagittal magnetic resonance imaging shows atlantoaxial dislocation, compression of the spinal cord by the odontoid process, and chiari malformation

**Fig. 5:**
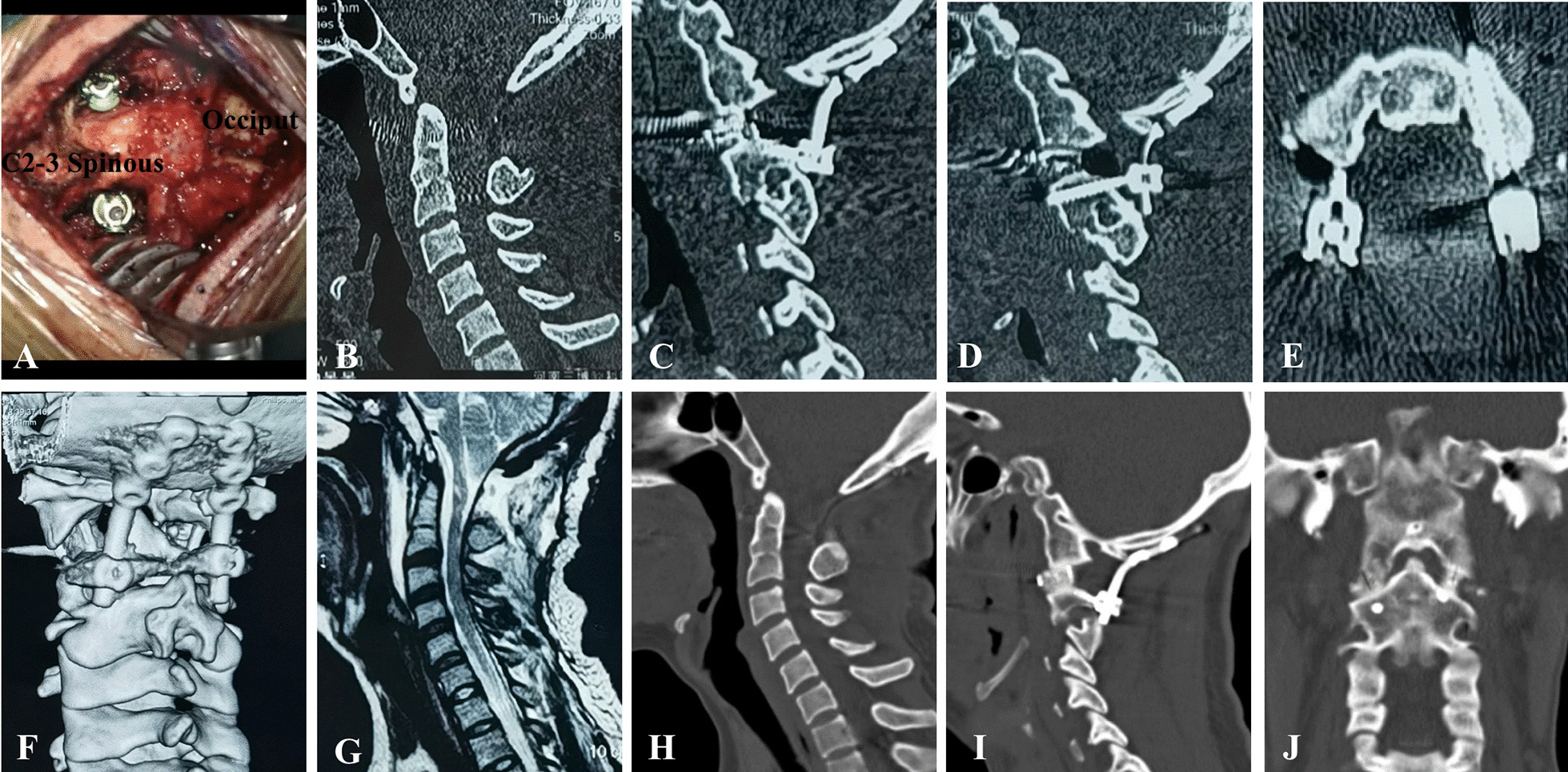
**A** After cage implantation, screws were inserted below the bilateral C2 articular surface. **B** Postoperative sagittal computed tomography (CT) showed that the odontoid process had been reduced and the cage was inserted anteriorly between the bilateral joints. **C**, **D**, **E**, **F** The insertion point of the C2 screw was located below the bilateral C2 articular surface and in a small area above the vertebral artery. The screw was inserted medially as far as possible. **G** Postoperative sagittal T2 magnetic resonance (MR) image showing that the ventral spinal cord compression was completely relieved, and the ventral spinal cerebrospinal fluid (CSF) signal was recovered. At 13 months after surgery, CT showed that the odontoid process remained in the reduced state (**H**) and the atlantoaxial interarticular bone bridge was formed (**I**, **J**)

The reduction in AAD and improvement of clinical symptoms were achieved for all patients in this study (Figs. 4, 5). We consider C2 subfacetal screws to be a neglected alternative to pedicle screws for the management of patients KFS. The C2 subfacetal screw technique is simple and safe. The dural membrane can be observed directly inside the insertion point, and the risk of spinal cord injury is low. The insertion point is located above the VA and there is no risk of VA injury. Furthermore, the C2 subfacetal screw has a long insertion depth and good fixation stability.

However, this study had some limitations. There were only seven patients included in the study. Studies involving more patients or a larger number of participants are needed to confirm the efficacy and safety of this procedure. In addition, during this study, the average internal height of the C2 vertebra of the seven patients was more than 4 mm and the VA was far from the superior articular surface; therefore, there was enough space for subfacetal screw insertion. However, if the internal height was small and the VA was closer to the superior articular surface of C2, then this technique would not be appropriate. Therefore, more studies are needed to confirm whether HRVA are caused by isthmus height loss in patients with KFS. Additionally, the follow-up time was short, not allowing for observation of the long-term effects of inserted subfacetal screws. Therefore, studies with longer follow-up duration are needed to sufficiently monitor possible long-term effects. The trajectory length of the subfacetal screws was smaller than that of pedicle screws; therefore, the biomechanical properties may be inferior to that of pedicle screws.

## Conclusions

When C2-3 fusion is associated with HRVA or pedicle stenosis, there is sufficient space below the C2 articular surface for screw placement. The C2 subfacetal screw is a viable alternative treatment for KFS with pedicle stenosis.

## Data Availability

Not applicable.
